# Comparison of Short-Arm Immobilization and Long-Arm Immobilization in Conservatively Managed Distal Radius Fractures: A Meta-Analysis and Systematic Review

**DOI:** 10.7759/cureus.55813

**Published:** 2024-03-08

**Authors:** Yash P Chaudhry, Genoveffa R Morway, Efstratios A Papadelis, Nikki A Doerr, Kenneth W Graf, Rakesh P Mashru, Henry J Dolch

**Affiliations:** 1 Orthopedic Surgery, Philadelphia College of Osteopathic Medicine, Philadelphia, USA; 2 Orthopedics and Traumatology, Cooper University Hospital, Camden, USA

**Keywords:** meta-analysis, systematic review, nonoperative treatment, immobilization, distal radius fracture

## Abstract

Distal radius fractures are often treated conservatively with immobilization. Immobilizing above the elbow limits forearm rotation, though recent literature has suggested the effects on radiographic or functional outcomes may be negligible. This systematic review and meta-analysis aimed to analyze the radiographic and functional outcome scores of distal radius fractures managed with short-arm (SA) immobilization and long-arm (LA) immobilization. An electronic systematic search was performed of the PubMed and EMBASE databases from inception to October 5, 2022. All randomized controlled trials (RCTs) involving patients with acute distal radius fractures undergoing nonoperative treatment (involving application/maintenance of immobilization) comparing above-elbow versus below-elbow constructs were included. The outcomes of interest were changes in radiographic parameters (loss of volar tilt [VT], radial height [RH], and radial inclination [RI]), loss of reduction, requirement for surgery, and patient-reported functional outcomes (Disabilities of the Arm, Shoulder, or Hand [DASH] or Quick DASH survey). The Cochrane Risk of Bias Tool 2.0 was used for study quality assessment. The effect size of the interventions was assessed using random effect models to calculate mean differences (MDs) for continuous variables and odds ratios (ORs) for categorical variables. Standardized mean difference (SMD) was calculated for patient-reported functional outcome scores. Nine studies involving 983 cases were included, including 497 SA and 486 LA. No statistically significant differences were observed with regards to VT (*P *= 0.83), RH (*P *= 0.81), RI (*P *= 0.35), loss of reduction (*P *= 0.33), requirement for surgery (*P *= 0.33), or patient-reported functional outcomes (*P *= 0.10). There was no difference in radiographic outcomes, need for surgery, or functional scores among patients treated with SA and LA immobilization. Utilizing SA immobilization is a safe option for conservative management of distal radius fractures and the benefits of mitigating complications associated with LA immobilization may supersede the theoretical limited forearm rotational stability observed with SA immobilization. Further study is required to determine the optimal method of SA immobilization.

## Introduction and background

Distal radius fractures are among the most common fragility fractures sustained in the elderly [1]. Historically, treatment of these injuries in the elderly population consisted of closed reduction and splint or cast immobilization with either above-elbow or below-elbow constructs [2-5]. Examples of above-elbow or long-arm (LA) immobilization, include sugar tong splints, posterior LA splints, and LA casts while below-elbow or short-arm (SA) constructs include SA splints, reverse sugar tong splints, and SA casts. Many surgeons advocate for immobilization constructs that extend proximal to the elbow to theoretically better control pronation and supination [1,6]. However, studies have reported that exposing the bony prominences of the elbow to plaster or other hard splint material for long periods increases the risk of pressure ulcers, especially in vulnerable populations such as demented, obtunded, or unreliable patients [1].

The optimal method of immobilization that provides fracture stability while allowing for patient comfort and function remains controversial [1-3]. Recent literature has challenged the necessity of including the elbow in the immobilization, as several studies have demonstrated no significant difference in outcomes between above-elbow immobilization and below-elbow constructs [3,7]. This study aimed to assess existing literature for randomized controlled trials and investigate our research question: Does SA immobilization offer similar radiographic parameters, requirement for operative intervention, and patient-reported functional outcomes as LA immobilization for conservatively managed distal radius fractures?

## Review

Methodology

Literature Search Strategy

A search was performed of all literature from inception to October 5, 2022, from the PubMed and EMBASE electronic databases involving the terms “distal radius” and “cast” or “splint.” MeSH and Emtree terms were used to enhance search sensitivity. This systematic review and meta-analysis was performed according to the guidelines set by the Preferred Reporting Items for Systematic Reviews and Meta-Analyses [8]. It was registered in the PROSPERO International Prospective Register of Systematic Reviews and can be found with the identifier CRD42022379897. No sources of funding were used for this research. 

Inclusion and Exclusion Criteria

All randomized controlled trials (RCTs) written in the English language involving patients >18 years old with acute distal radius fractures with plans for nonoperative treatment (involving the application and maintenance of immobilization constructs) were considered for inclusion. Only studies that compared above-elbow constructs versus below-elbow constructs were included.

Study Selection

Studies were initially screened using titles and abstracts, followed by a full-text review of relevant studies. All screening was performed independently by two authors. Any disagreements were brought to the attention of the senior author for resolution on an as-needed basis.

Data Extraction

Data extraction was performed manually - variables extracted from the included studies included year and country of publication, patient demographics (age, sex, body mass index [BMI], and hand dominance), interventions being compared (types of immobilization), inclusion and exclusion criteria, length of follow up, fracture characteristics (dorsal comminution, articular involvement, ulnar styloid fracture, and prereduction radiographic characteristics, including volar tilt [VT], radial height [RH], and radial inclination [RI]), and follow-up outcomes (radiographic loss of reduction, post-reduction radiographic characteristics, the requirement for operative intervention, and patient-reported functional outcomes, such as Disabilities of the Arm, Shoulder, and Hand [DASH] score or validated Quick DASH score).

Risk of Bias

The Cochrane Risk of Bias Tool 2.0 was used for the quality assessment of included studies [9]. Five domains were examined: randomization, deviations from the intended intervention, missing data, measurement of outcomes, and selection of reported results. For each domain, a risk score of *low*, *some concern*, or *high* was assigned. The Robvis tool was used to generate a risk-of-bias chart to summarize quality appraisal [10].

Data Analysis

Continuous variables were collected and presented in the form of mean and standard deviation. When these values were not available in the included studies, they were imputed using the methodology established in the Cochrane Handbook for Systematic Reviews of Interventions [11]. Categorical variables were presented as counts with percentages. The effect size of the interventions was assessed using random effect models to calculate mean differences (MDs) for continuous variables and odds ratios (ORs) for categorical variables. Standardized mean difference (SMD) was calculated for patient-reported functional outcome scores to allow for standardization between different scoring methods of the DASH and Quick DASH surveys. Heterogeneity was reported with the *I*^2^ statistic. Forest plots were generated for each study outcome using Review Manager Software Version 5.4.1 (The Nordic Cochrane Center, The Cochrane Collaboration, 2020, Copenhagen).

Outcome Measures

The primary outcomes of interest in this review were the change from pre-reduction to post-reduction for the radiographic values of VT, RH, and RI, radiographic loss of reduction, requirement for operative intervention, and patient-reported functional outcome score at follow-up.

Results

Study Selection 

**Figure 1:**
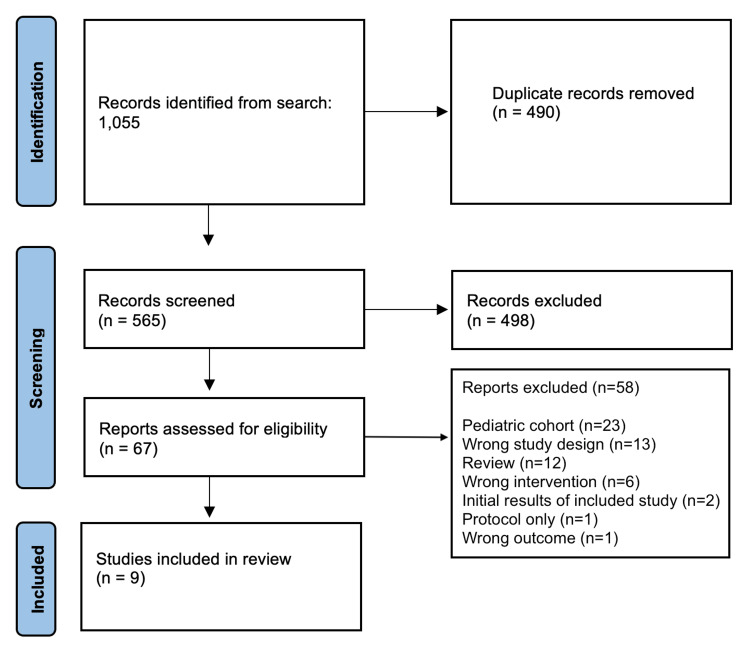
PRISMA flow diagram demonstrating the selection process of reviewed articles. PRISMA, Preferred Reporting Items for Systematic Reviews and Meta-Analysis

A total of 1,055 articles were identified related to the topic of this review of which 490 were duplicate records. Of the 565 abstracts included at the initiation of screening, 67 were identified for full-text review. Of these reports, 23 (40%) included pediatric patients, 13 (22%) had an incorrect study design, 12 (21%) were review articles, and 6 (10%) investigated the wrong intervention, including wrist positions in splints (*n* = 2, 3%), external fixation compared to cast immobilization (*n* = 1, 2%), sugar tong splinting compared to cast immobilization (*n* = 1, 2%), LA immobilization compared to closed reduction and percutaneous pinning or SA immobilization (*n* = 1, 2%), and initial sugar tong immobilization followed by conversion to SA or LA immobilization (*n* = 1, 2%). Two reports presented the initial results of articles included in the review, another report solely described the protocol, and one reported an incorrect outcome (Figure 1).

Nine studies, all RCTs, were selected for final inclusion. The review includes 983 fractures from several different countries such as the United States, Turkey, Italy, Spain, Canada, Iran, and Brazil. Patients were mostly aged over 60 years, with a greater proportion of females compared to males. BMI was not recorded in any of the studies. Three studies included information about the dominant hand, and the length of follow-up varied from seven days to one year. Patient characteristics and study characteristics, including inclusion and exclusion criteria were summarized in Table 1.

**Table 1:** Study characteristics of nine randomized controlled trials. BMI, body mass index; NR, not reported; SA, short-arm immobilization construct; LA, long-arm immobilization construct; AO, Arbeitsgemeinschaft für Osteosynthesefragen; DR, distal radius; GA, Gustillo-Anderson; NV, neurovascular; VT, volar tilt; RI, radial inclination; ORIF, open reduction and internal fixation; RH, radial height

Author, Year	Country	N	Age (yr)	Female (%)	BMI	Dominant hand	Compared constructs	Inclusion/exclusion criteria	Length of follow-up
Bong et al. (2006) [12]	United States	85	64	69	NR	NR	SA radial gutter LA: sugar tong	Inclusion: closed fractures, age > 18 years; exclusion: nondisplaced fractures	7-10 days
Çamur et al. (2021) [4]	Turkey	127	58	68	NR	91%	SA: reverse sugar tong LA: below arm cast	Inclusion: AO type A/B DR fractures age > 18 years; exclusion: AO type C DR fractures, comminuted fractures, surgically treated, GA type 2 or 3 open fractures, prior hand or wrist surgery, additional ipsilateral extremity fractures, pathological fractures, and cognitive deficits limiting study compliance	7-10 days, then 3 weeks, 5-6 weeks (immobilization removed), 12 weeks, and 1 year
Caruso et al. (2019) [13]	Italy	74	71	90	NR	NR	SA: short cast; LA: long cast	Inclusion: age > 18 years, extra-articular fractures with dorsal displacement; exclusion: open fractures, extra-articular fractures with volar displacement, and surgical treatment required	7-10 days, 4 weeks, 12 weeks
Dib et al. (2022) [14]	Turkey	280	70	86	NR	NR	SA: below-elbow cast; LA: above-elbow cast	Inclusion: age > 18 years, nonoperative treatment candidates, displaced fracture requiring manipulation; exclusion: skeletal immaturity, non-displaced fracture, open fracture, NV injury, bilateral fracture, additional ipsilateral extremity fracture	7 days, 35 days (immobilization removed)
Gamba et al. (2017) [15]	Spain	72	77	96	NR	NR	SA: below-elbow cast; LA: above-elbow cast	Inclusion: VT ³ 0 deg, RI ³ 20 deg, articular step off <2 mm; exclusion: volar displacement, metaphyseal extension, open fractures, bone dysplasia, prior fractures, cognitive impairment, and surgical treatment	1 week, 3 weeks, and 6 weeks (immobilization removed)
Grafstein et al. (2010) [16]	Canada	61	58	82	NR	NR	SA: volar dorsal splint; LA: modified sugar tong	Inclusion: age > 18 years, displaced fracture; exclusion: open fracture, previously displaced fracture of either wrist, neuromuscular deficits, cerebrovascular events, concurrent carpal fracture/dislocation, fracture requiring ORIF, skin reaction to immobilization material, Smith/Barton/chauffeur fractures, NV injury, bilateral DR fracture, nondisplaced fracture, and no reduction	1 week, 3-4 weeks
Mahmoudi et al. (2019) [17]	Iran	80	50	58	NR	NR	SA: thumb spica splint; LA cast	Inclusion: nondisplaced fracture; exclusion: age <20 or >70 years, unstable fractures, intra-articular fractures, >20 degrees of dorsal angulation, fractures associated with other organ damage, open fracture, previous severe deformity of limb, diabetes, and severe osteoporosis	4 weeks, 6 weeks (immobilization removed), 12 weeks
Okamura et al. (2021) [18]	Brazil	128	62	69	NR	98%	SA radial cast; LA: above-elbow cast	Inclusion: displaced and reducible fractures (defined by loss of RH >2 mm, loss of RI >4 degrees, dorsal tilt >10 degrees, positive ulnar variance loss >3 mm, intra-articular step off >2 mm, or carpal malalignment); exclusion: open fractures, bilateral fractures, associated tendon/NV injury, presentation >7 days from injury, associated carpal fractures, required ORIF, prior history of degenerative/traumatic disorder of either wrist, any disease/lesion precluding immobilization application, and cognitive deficit	1 week, 2 weeks, 3 weeks, 4 weeks (LA transitioned to SA), 6 weeks (immobilization removed), 8 weeks, 12 weeks, and 24 weeks
Stevens et al. (2022) [19]	United States	89	57	82	NR	31%	SA: clamshell splint; LA: sugar tong splint	Inclusion: DR fracture requiring closed reduction; exclusion: age < 18, concomitant radial/ulnar shaft fracture, intra-articular fracture, open fracture, requiring ORIF	1-2 weeks, 6 weeks

Risk-of-Bias Analysis

**Figure 2:**
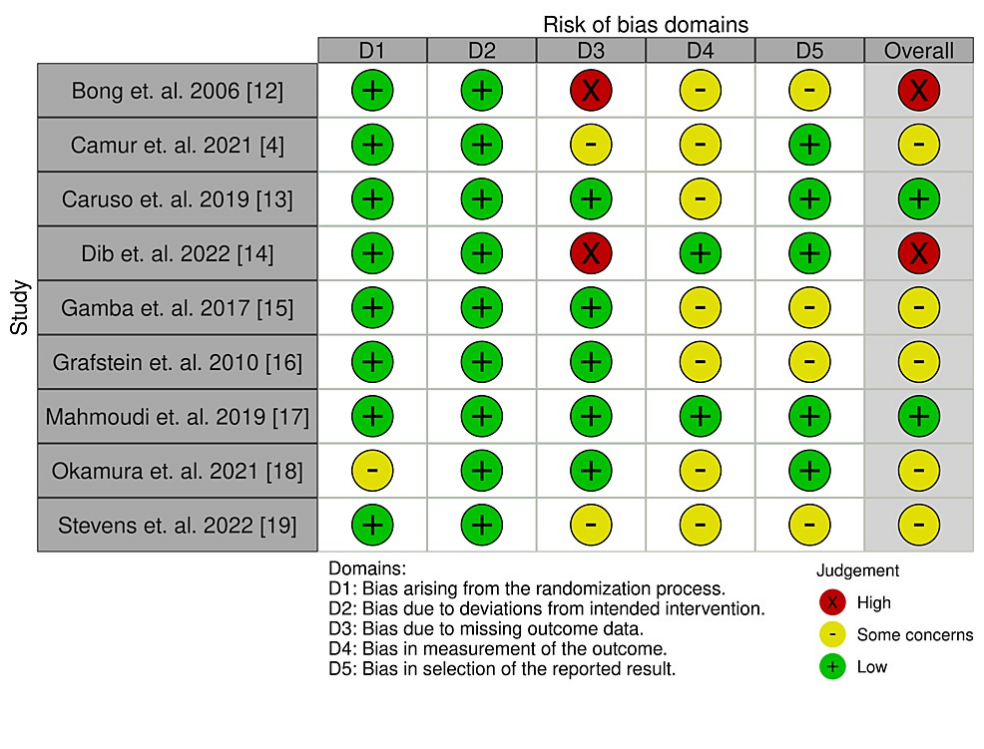
Risk-of-bias quality appraisal summary.

**Figure 3:**
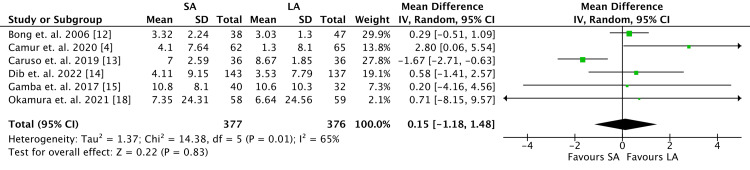
Volar tilt outcomes. SA, short arm; LA, long arm; SD, standard deviation; IV, weighted mean difference; CI, confidence interval; Chi^2^, chi-square statistic; df, degrees of freedom; *I*^2^, *I*-square heterogeneity statistics; *Z*, *Z*-statistics

Quality appraisal of the included studies was performed using the Cochrane Risk of Bias tool (Figures 2-3). Nine studies described their methods of randomization in which one was inexplicit in expanding on the process of randomization. All studies demonstrated a low risk of deviation from the intended intervention. Two studies raised concerns regarding missing outcome data and two were at high risk of bias; one failed to report radiographic measurements used for a primary outcome, while one lacked sufficient patient participation for a secondary outcome. Seven studies demonstrated bias in the measurement of outcomes, while there were some concerns about selection bias of results for four studies. 

Radiographic Outcomes 

Six of nine RCTs measured radiographic outcomes, thus 753 patients were analyzed (Figure 3). There were no differences between the two cohorts with regards to change in VT (MD 0.15, 95% confidence interval [CI] -1.18 to 1.48; *P* = 0.83), although the heterogeneity was high (*I*^2 ^= 65%).

**Figure 4:**
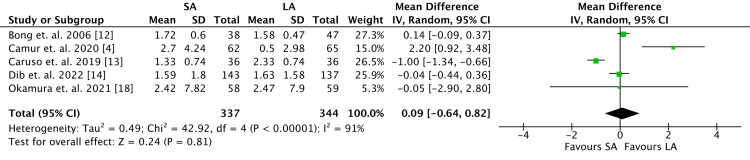
Radial height outcomes. SA, short arm; LA, long arm; SD, standard deviation; IV, weighted mean difference; CI, confidence interval; Chi^2^, chi-square statistic; df, degrees of freedom; *I*^2^, *I-*square heterogeneity statistics; *Z*, *Z*-statistics

Five studies reported outcomes for RH, including a total of 681 patients (Figure 4). Among the reported data, there were no differences in change in RH between the two cohorts (MD 0.09; 95% CI -0.64 to 0.82; *P* = 0.81).

**Figure 5:**
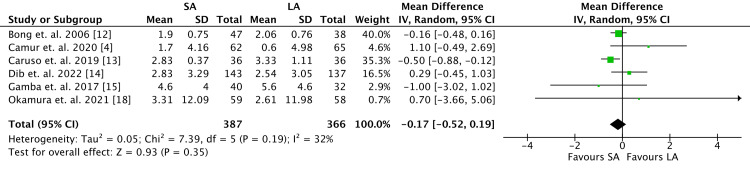
Radial inclination outcomes. SA, short arm; LA, long arm; SD, standard deviation; IV, weighted mean difference; CI, confidence interval; Chi^2^, chi-square statistic; df, degrees of freedom; *I*^2^, *I*-square heterogeneity statistics; *Z*, *Z*-statistics

Six studies included RI in their reported data with 753 patients (Figure 5). Similarly, there was no difference between the two cohorts with regard to change in RI (MD -0.17, 95% CI -0.52 to 0.19; *P* = 0.35). Low heterogeneity was demonstrated (*I*^2 ^= 32%). 

**Figure 6:**
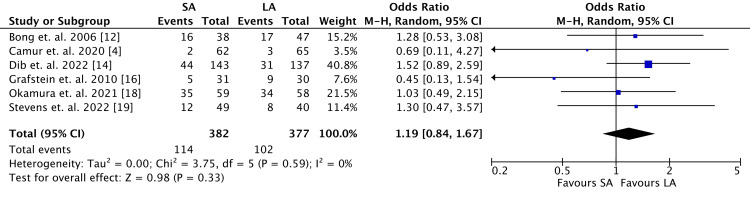
Loss-of-reduction outcomes. SA, short arm; LA, long arm; SD, standard deviation; IV, weighted mean difference; CI, confidence interval; Chi^2^, chi-square statistics; df, degrees of freedom; *I*^2^, *I*-square heterogeneity statistics; *Z*, *Z*-statistics

The rate of loss of reduction was higher in the SA cohort, although not statistically significant (SA: 113, 30%; LA: 103, 27%; OR 1.19, 95% CI 0.84-1.67; *P* = 0.33) (Figure 6). Heterogeneity was very low among data points (*I*^2 ^= 0%). Data for loss of reduction was reported by six RCTs and included 759 patients. 

**Figure 7:**
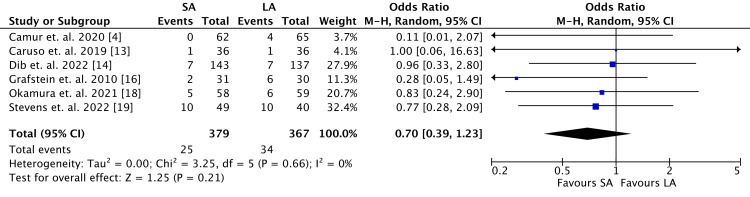
Requirement for operative intervention outcomes. SA, short arm; LA, long arm; SD, standard deviation; IV, weighted mean difference; CI, confidence interval; Chi^2^, chi-square statistics; df, degrees of freedom; *I*^2^, *I*-square heterogeneity statistics; *Z*, *Z*-statistics

The SA cohort demonstrated a lower rate of the requirement for operative intervention (SA: 25, 6.6%; LA: 34, 9.3%; OR 0.70, 95% CI 0.39-1.23; *P* = 0.21) (Figure 7), although no statistically significant difference was observed. There were 746 patients reported by six studies in which heterogeneity was calculated as very low (*I*^2 ^= 0%).

Functional Outcomes 

**Figure 8:**
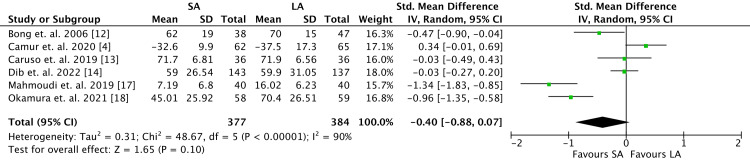
Functional outcomes represented as Disabilities of the Arm, Shoulder, or Hand (DASH) or Quick DASH survey scores. SA, short arm; LA, long arm; SD, standard deviation; IV, weighted mean difference; CI, confidence interval; Chi^2^, chi-square statistics; df, degrees of freedom; *I*^2^, *I*-square heterogeneity statistics; *Z*, *Z*-statistics

Six of nine RCTs were analyzed and included patient-reported upper extremity function through DASH scores (Figure 8). One study reported radiographic parameters only and lacked functional outcome scores, while two studies reported incomplete DASH scores. There were no observed differences with regard to patient-reported upper extremity function between the two groups, with 377 patients included in the SA cohort and 384 patients included in the LA cohort (SMD -0.40, 95% CI -0.88 to 0.07; *P* = 0.10).

Discussion 

Distal radius fractures are among the most common fractures in adult populations [20]. Conservative management in the form of closed reduction and immobilization is commonly used in elderly patients as it has shown equal efficacy when compared to operative fixation with regard to wrist pain and function [21]. The purpose of this study was to investigate the question: Does SA immobilization offer similar outcomes to LA immobilization for conservatively managed distal radius fractures? No statistically significant differences were found with regard to changes in radiographic parameters, requirement for operative treatment, or subjective patient-reported functional outcomes. The results of this systematic review and meta-analysis suggest that SA immobilization is equally effective as LA immobilization.

SA immobilization may offer several benefits, particularly in elderly patients. Osteoporotic patients who are at the greatest risk of sustaining injuries such as distal radius fractures are more likely to have dementia or other cognitive impairments affecting their mental status [22,23]. In such patient populations, avoiding pressure ulcers secondary to immobilization material beneath the bony prominences of the elbow is paramount [24,25]. This is also true of patients who are obtunded or sedated and cannot report symptoms such as pain or pressure [26]. Furthermore, these patients are often frail or cachectic and may not be able to tolerate the weight of LA constructs prompting the use of a sling [27]. Previous studies have demonstrated that LA immobilization allows for better control of supination and pronation when compared to SA immobilization [6]; however, the results of this review suggest that the clinical significance of controlling for supination and pronation in certain patient populations may be negligible.

Several of the SA constructs included in this review used a single piece of plaster or fiberglass. For example, Çamur et al. [4] investigated the use of reverse sugar tong splints compared to below arm cast for distal radius fractures. Because the volar and dorsal slabs in the reverse sugar tong splint remain connected by a bridge draped over the first webspace, theoretically they can maintain the forearm in the same neutral position throughout immobilization. However, even in SA constructs that consisted of two separate plaster slabs, such as the ones used in the study conducted by Stevens et al. [19], there were no differences in radiographic parameters or requirements for surgery between the SA and LA constructs. The current study demonstrated a higher rate of conversion to operative treatment in the LA construct cohort, although this was not statistically significant.

The results of this review are similar to those reported in the literature. In a systematic review of 22 RCTs and retrospective studies comparing eight different immobilization methods used in distal radius fractures, Jamnik et al. [28] found no significant differences in radiographic parameters or functional outcomes across any of the immobilization constructs. Although they were able to provide a more granular approach, including the examination of splint versus casts, radial gutter versus spica constructs, or even simple dorsal splint constructs, they were limited in their ability to assess the role of immobilizing the elbow. In a similar systematic review, Saka et al. [27] examined 10 RCTs comparing immobilization methods of distal radius fractures and reported a statistically significant difference in DASH scores favoring SA immobilization, but the difference was not clinically significant. They also reported no differences in radiographic outcomes or treatment failure (either loss of reduction or requirement for surgical intervention). The present study supplements the data reported in these reviews by incorporating articles published within the last two years, including studies conducted by Dib et al. [14] and Stevens et al. [19], which collectively constitute over one-third of the cases included in this review. In summary, the current literature leans toward equivocal results regarding the use of SA versus LA immobilization constructs.

Limitations

The most prominent limitation of the current study is the heterogeneity of the included studies. The use of different immobilization constructs introduces variability between studies. Additionally, the decision to proceed with operative intervention is dependent on surgeon preference, who may have varying thresholds for converting to surgical treatment. This is evident in comparing rates of operative treatment between studies, ranging from 5% as reported by Dib et al. [14] to 22% as reported by Stevens et al. [19]. Another limitation is the difference in methods used to describe and classify fracture patterns. Several studies divided fractures into stable versus unstable injuries, but different criteria were used for classification. Further breakdown of fracture characteristics within the included studies may have provided some additional benefit as control of prono-supination may be of greater benefit in cases involving fractures with higher energy mechanisms and soft tissue disruption. Similarly, loss of reduction was based on slightly different criteria in the included studies. In contrast, the change in radiographic parameters between the two cohorts is much more generalizable as these measurements are more reproducible between studies. One of the strengths of this study was the restriction to only include RCTs, thereby minimizing selection bias.

## Conclusions

The results of this systematic review and meta-analysis show that the efficacy of SA immobilization is comparable to LA immobilization in the conservative management of distal radius fractures. This is particularly true for patients who may benefit from avoiding plaster or cast material around the elbow, such as patients with altered mental status and dementia. There were no statistically significant differences in change in radiographic parameters, requirement for operative treatment, or subjective patient-reported functional outcomes. These results should be interpreted carefully as certain study limitations exist. Further high-quality studies are warranted to characterize the optimal fracture patterns amenable to motion at the elbow and SA immobilization.
